# Accumulation of LDL/ox-LDL in the necrotic region participates in osteonecrosis of the femoral head: a pathological and *in vitro* study

**DOI:** 10.1186/s12944-021-01601-x

**Published:** 2021-11-25

**Authors:** Xin-Yuan Wang, Tian-Le Ma, Kang-Ning Chen, Zhi-Ying Pang, Hao Wang, Jun-Ming Huang, Guo-Bin Qi, Chen-Zhong Wang, Zeng-Xin Jiang, Lin-Jing Gong, Zhe Wang, Chang Jiang, Zuo-Qin Yan

**Affiliations:** 1 Present Address: Department of Orthopaedics, West China Hospital, West China Medical School, Sichuan University, Chengdu, 610041 Sichuan Province China; 2 Department of Orthopaedics, Zhongshan Hospital, Fudan University, 180 Fenglin Road, Shanghai, 200032 China; 3 Department of Respiratory and Critical Care Medicine, West China Hospital, Sichuan University, Chengdu, Sichuan 610041 China

**Keywords:** Osteonecrosis of the femoral head, Low-density lipoprotein, Oxidized low-density lipoprotein, Osteocyte, Hypoxia

## Abstract

**Background:**

Osteonecrosis of the femoral head (ONFH) is a common but intractable disease that appears to involve lipid metabolic disorders. Although numerous studies have demonstrated that high blood levels of low-density lipoprotein (LDL) are closely associated with ONFH, there is limited evidence to explain the pathological role of LDL. Pathological and *in vitro* studies were performed to investigate the role of disordered metabolism of LDL and oxidized LDL (ox-LDL) in the femoral head in the pathology of ONFH.

**Methods:**

Nineteen femoral head specimens from patients with ONFH were obtained for immunohistochemistry analysis. Murine long-bone osteocyte Y4 cells were used to study the effects of LDL/ox-LDL on cell viability, apoptosis, and metabolism process of LDL/ox-LDL in osteocytes in normoxic and hypoxic environments.

**Results:**

In the pathological specimens, marked accumulation of LDL/ox-LDL was observed in osteocytes/lacunae of necrotic regions compared with healthy regions. *In vitro* studies showed that ox-LDL, rather than LDL, reduced the viability and enhanced apoptosis of osteocytes. Pathological sections indicated that the accumulation of ox-LDL was significantly associated with impaired blood supply. Exposure to a hypoxic environment appeared to be a key factor leading to LDL/ox-LDL accumulation by enhancing internalisation and oxidation of LDL in osteocytes.

**Conclusions:**

The accumulation of LDL/ox-LDL in the necrotic region may contribute to the pathology of ONFH. These findings could provide new insights into the prevention and treatment of ONFH.

**Supplementary Information:**

The online version contains supplementary material available at 10.1186/s12944-021-01601-x.

## Background

Osteonecrosis of the femoral head (ONFH) is a common but intractable orthopaedic disease that mainly affects younger people. Trauma, glucocorticoids (GCs), and alcohol are the causal agents in most cases of ONFH. Although global epidemiological studies have not been conducted, there are about 10000 to 20000 new cases of ONFH per year in the United States [1]. In China, it is estimated that 8.12 million patients have non-traumatic ONFH [2]. ONFH is a multifactorial disorder and the underlying pathophysiologic mechanisms remain unclear [3].

Circulating and local lipid metabolic disorders are crucial in ONFH, especially in cases associated with GC or alcohol abuse [3]. The conventional theory assumes that disordered lipid metabolism elicits its effects through two pathways, both of which ultimately lead to impaired blood supply [4]. The first pathway involves the promotion of differentiation and maturation of adipocytes from bone marrow stromal cells (BMSCs) and pre-adipocytes, which may block the medullary blood supply. The second pathway involves altered levels of circulating lipids, which may form fat microemboli in the microvessels of the femoral head.

Low-density lipoprotein (LDL), known as bad cholesterol, is a vehicle for transporting cholesterol. High blood levels of LDL are associated with idiopathic ONFH [5] and osteonecrosis in patients with systemic lupus erythematosus [6]. An epidemiological survey of 30,030 respondents revealed that high LDL levels are associated with increased risk of non-traumatic ONFH [2]. Furthermore, high LDL is an independent risk factor of postoperative ONFH in those with femoral neck fractures [7]. However, there is limited evidence to explain the pathological role of LDL in ONFH, and local LDL metabolism in the femoral head is poorly understood. LDL is a complex particle that is sensitive to oxidation and readily forms oxidized low-density lipoprotein (ox-LDL). Because ox-LDL contains various oxidized proteins and lipids with potential oxidative toxicity, it can trigger apoptosis of many cell types and plays a key role in atherosclerosis [8]. Since ONFH shares a number of risk factors with atherosclerosis [2], understanding the role of ox-LDL in atherosclerosis may shed new light on ONFH.

In this work, pathological and *in vitro* studies were performed to investigate the role of disordered metabolism of LDL and ox-LDL in the femoral head in the pathological process of ONFH. Preliminary studies were also performed to explore the reasons for the accumulation of LDL/ox-LDL in the femoral head.

## Methods

### Reagents and antibodies

LDL and ox-LDL were obtained from Solarbio (Beijing, China). Primary antibodies against cleaved-caspase3, Bax, β-actin, and secondary antibody for western blotting were obtained from Cell Signaling Technology (Danvers, USA), and those against LDL and ox-LDL were obtained from Bioss (Beijing, China) and Biorbyt (Cambridge, UK), respectively.

### Femoral head specimens and immunohistochemistry (IHC)

Nineteen femoral head specimens were obtained from consecutive patients with ONFH who underwent total hip arthroplasty (THA) or core decompression with bone grafting at Zhongshan Hospital, Fudan University, Shanghai between May 2019 and December 2019. The inclusion and exclusion criteria, and their clinical characteristics (Supplemental Table 1) are provided in the Supplemental Materials. The fresh femoral head specimens were fixed in 4% paraformaldehyde for 14 days, and decalcified with 10% EDTA. The whole specimen obtained from hip-preserving surgery or a rectangular specimen from the femoral head obtained by THA were dehydrated and embedded in paraffin. Each specimen contained necrotic and healthy regions. For IHC, the specimens were cut for 5-μm-thick sections, and incubated with primary antibodies against LDL (1:200) and ox-LDL (1:100) separately. Representative images were recorded by optical microscope (IX73, Olympus, Tokyo, Japan) and representative sections were digitally scanned using Pannoramic DESK (3D HISTECH, Budapest, Hungary).

The percentage of empty lacunae was analysed manually, and the necrotic and healthy regions were distinguished as described previously [9]. The percentage of LDL/ox-LDL-positive osteocytes/lacunae among all osteocytes/lacunae, and the percentage of empty lacunae among LDL/ox-LDL-positive lacunae were calculated manually. The intensity of osteocyte/lacuna staining was graded as from 0 (negative) to 3 (intense). According to a previous study [10], the immunoreactive score (IRS) for each microscopic field was obtained by multiplying the percentage of immunopositive osteocytes/lacunae (×100%) by staining intensity grading. The quantity and diameter of the medullary microvessels in the necrotic region were measured on the scanned sections. Ten random fields (400×) of both necrotic and healthy regions in each section were used for the analysis.

### Cell culture

Murine long-bone osteocyte Y4 (MLO-Y4) osteocyte-like cell line is one of the most widely used osteocyte models *in vitro* [11]. The MLO-Y4 cell line and macrophage cell line RAW 264.7 were acquired from Chinese Academy of Sciences Cell Bank. MLO-Y4s were cultured in α-MEM medium with 5% fetal bovine serum (FBS) and 5% calf serum (CS), and RAW 264.7s were cultured in DMEM medium with 10% FBS, both at 37°C with 21% O_2_ and 5% CO_2_. The hypoxic environment was set by adjusting the gas concentrations to 5% CO_2_ and 1% O_2_ using N_2_. The culture media were changed every 1 or 2 days.

### Cell viability assay

MLO-Y4s were planted at a density of 1×10^4^ cells/mL (100μL/well) and cultured for 24h for attachment. They were treated with varying concentrations of LDL or ox-LDL (0, 1, 2.5, 5, 10, 25, 50, or 100μg/mL) for 12, 24, or 48h. Cell viability was tested with Cell Counting Kit-8 (CCK-8, AbMole, Shanghai, China). Microplate reader (Thermo Fisher Scientific, Waltham, USA) was used for optical density measurement at 450 nm.

### Western blotting

After treating MLO-Y4s with LDL or ox-LDL (0, 5, 10, 25, or 50μg/mL) for 24 or 48h, the total protein was exacted with RIPA lysis buffer (Beyotime, Shanghai, China). Western blotting was conducted to quantify the protein levels of cleaved-caspase3, Bax, and β-actin. Briefly, the proteins were separated by SDS-PAGE and transferred to PVDF membranes, and then blocked using 5% bovine serum albumin (BSA) for 1h. After incubation with the primary antibodies at 4°C overnight and the secondary antibody for 1h at room temperature, the membranes were detected by enhanced chemi-luminescence (Yeasen, Shanghai, China) and quantitatively analysed with ImageJ software (Bethesda, Rockville, USA).

### Immunofluorescence assay

MLO-Y4s were planted in 24-well plates and cultured for 24h for attachment. Based on the dose-dependent effects of LDL and ox-LDL on cell viability, a dose of 25μg/mL was selected for subsequent LDL and ox-LDL metabolism experiments. The cells were incubated with LDL or ox-LDL for 0, 6, 12, or 24h, fixed with 4% paraformaldehyde and blocked with 1% BSA. The MLO-Y4s were incubated with antibodies against either LDL (1:200) or ox-LDL (1:200) at 4°C overnight, and then with an Alexa Fluor 594-conjugated fluorescently labelled secondary antibody at room temperature for 1h. 4′,6-diamidino-2-phenylindole (Beyotime) was used for nucleus staining and Olympus IX71 microscope was used for image recording. The percentage of LDL/ox-LDL-positive cells were determined in five random fields (200×).

### Quantitative real-time polymerase chain reaction (qRT-PCR)

Total RNA was extracted from MLO-Y4 or RAW 264.7 cells using Trizol (Invitrogen, Carlsbad, USA) and reverse-transcribed using PrimeScrip RT Master Mix (Takara, Kyoto, Japan). Primers for *GAPDH* (B661304) were purchased from and other primers were designed by Sangon (Shanghai, China; Supplemental Table 2). qRT-PCR was conducted with a qRT-PCR system (Bio-Rad CFX96, Applied Biosystems, Carlsbad, USA). The mRNA expression was measured in triplicate and normalised to *GAPDH*.

### Malondialdehyde (MDA) assay

MLO-Y4s were treated with LDL or ox-LDL at the concentration of 25μg/mL for 0, 6, 12, or 24h. The culture supernatant and cell cytoplasm obtained after lysis with RIPA lysis buffer (Beyotime) were collected. A commercial lipid peroxidation assay kit (S0131S, Beyotime) was used to measure the MDA levels in the culture supernatant and osteocytes.

### Statistical analysis

Statistical analyses were conducted using SPSS v25.0 (IBM, Armonk, USA). Between-group comparisons were made using Student’s *t* test, the Mann-Whitney *U* test, or the Kruskal-Wallis test, as appropriate. Comparisons of necrotic and healthy regions or different sections of individual specimens were made using paired *t*-test or Wilcoxon’s paired test. Correlation analyses were performed using Spearman’s correlation coefficients. All values are presented as the mean ± standard deviation (SD) of at least 3 independent experiments and *P* < 0.05 was regarded as statistically significant.

## Results

### Although LDL and ox-LDL accumulated in the necrotic region, the empty bone lacunae were more likely to accumulate ox-LDL.

**Fig. 1:**
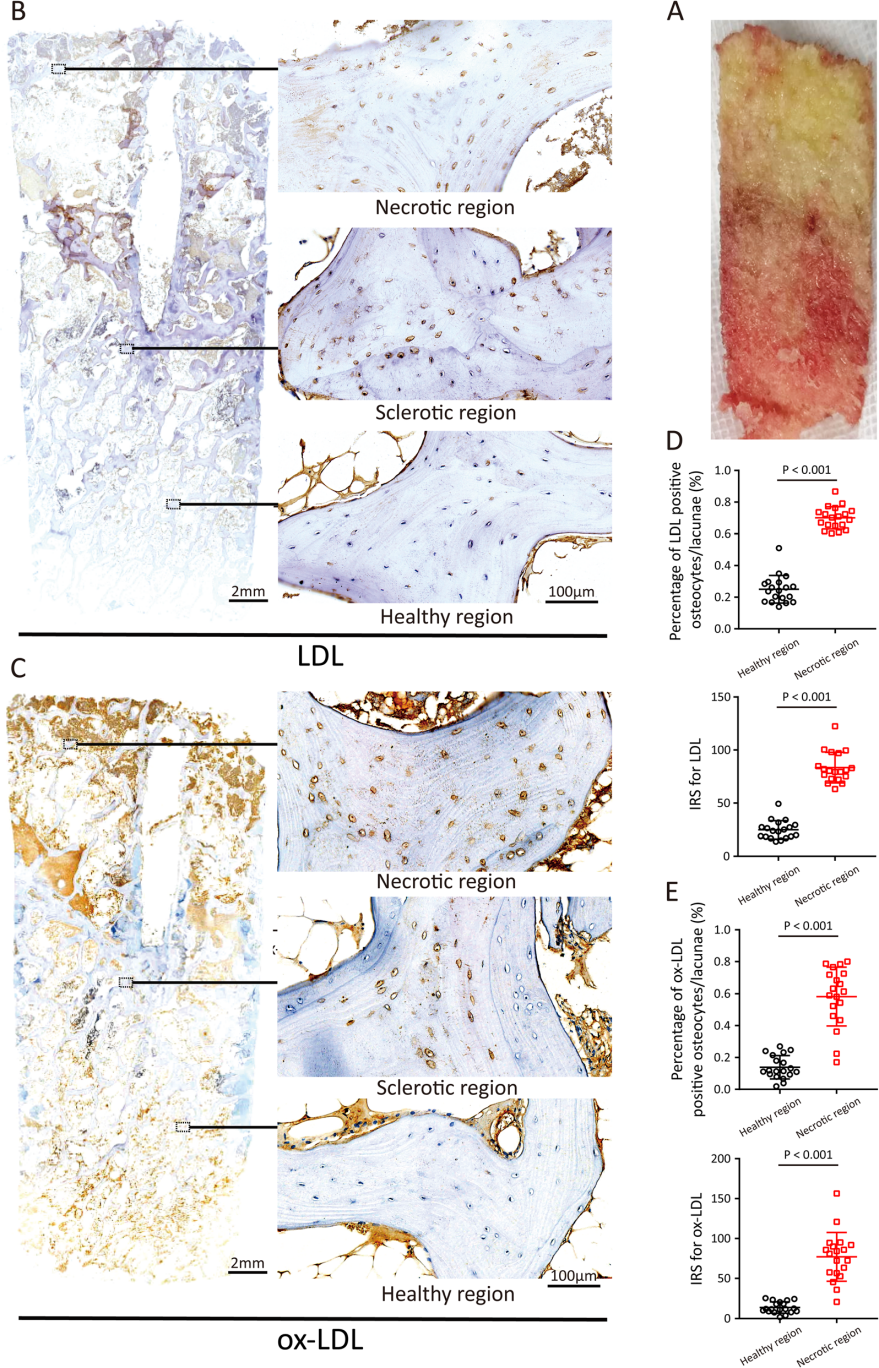
LDL and ox-LDL accumulated in the necrotic region of ONFH. **A** A representative femoral head specimen from the patient underwent hip-preserving surgery. **B** IHC staining of LDL on a representative pathological section of ONFH. **C** IHC staining of ox-LDL on a representative pathological section of ONFH. **D** The percentage of LDL-positive osteocytes/lacunae and IRS in necrotic and healthy regions. **E** The percentage of ox-LDL-positive osteocytes/lacunae and IRS in necrotic and healthy regions. Ten random fields (400×) of both necrotic and healthy regions in each specimen were observed and calculated for analysis. Data are represented as the mean ± SD of 19 specimens, and comparisons are made using paired *t*-test or Wilcoxon’s paired test

Figure 1A shows distinct fat accumulation in the necrotic region in gross specimens. Representative pathological sections of ONFH that contained necrotic, sclerotic, and healthy regions are shown in Figures 1B and C. In necrotic regions, the dead trabeculae were disorganised and broken, and there was extensive loss of living osteocytes in the bone lacunae. The necrotic and healthy regions were separated by sclerotic regions, where large numbers of dead and living trabeculae were joined. In healthy regions, the trabeculae showed a normal arrangement and the medullary tissue was intact.

Figures 1B and C show that LDL and ox-LDL accumulated in necrotic regions, in stark contrast to the healthy regions. The dead and living osteocytes in the necrotic region were generally positive for LDL and ox-LDL unlike those in the healthy region. The quantitative analysis of the IHC sections showed that the percentage of LDL/ox-LDL-positive osteocytes/lacunae and the IRS of the necrotic region were significantly greater than those of healthy regions (*P* < 0.001, Figures 1D and E).

**Fig. 2:**
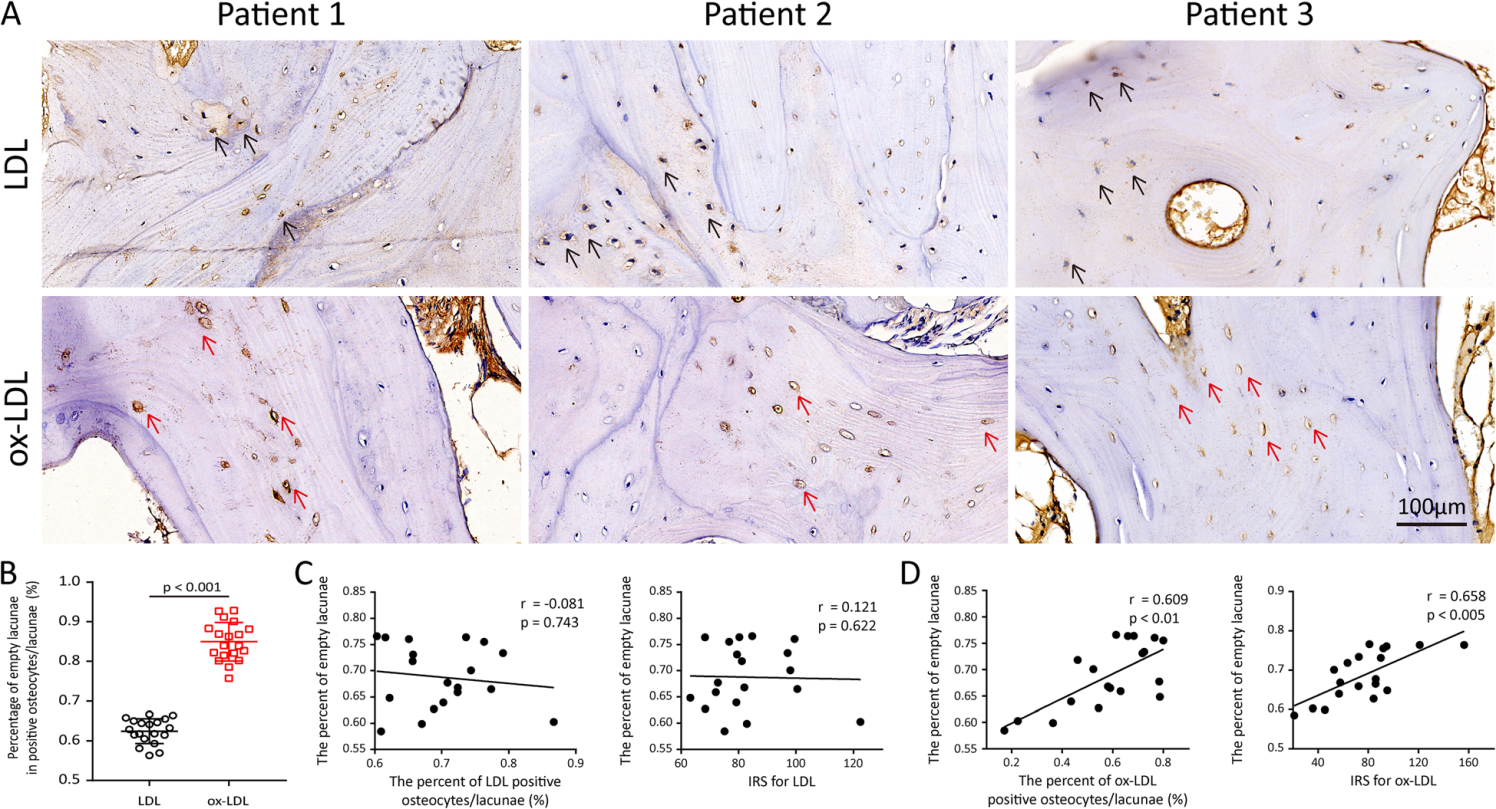
Empty bone lacunae were more likely to be ox-LDL-positive rather than LDL-positive. **A** Representative IHC staining images of the junctional region of living and dead trabeculae. Ox-LDL was more likely to accumulate in empty lacunae (red arrows) when compared with LDL (black arrows). **B** The percentage of empty bone lacunae among LDL-positive and ox-LDL-positive osteocytes/lacunae in the necrotic region. **C** Correlation between the percentage of empty lacunae in the necrotic region with the percentage of LDL-positive osteocytes/lacunae and IRS for LDL. **D** Correlation between the percentage of empty lacunae in the necrotic region with the percentage of ox-LDL-positive osteocytes/lacunae and IRS for ox-LDL. Ten random fields (400×) in each specimen were observed and calculated for analysis. Data are represented as the mean ± SD of 19 specimens. Between-group comparisons are made using Student’s *t* test, and Spearman’s correlation coefficients are used for correlation analysis

Although LDL and ox-LDL accumulated in the bone lacunae in the necrotic regions, there were differences in the distribution of immunopositive lacunae. The representative images of the junctions between the living and dead trabeculae revealed that the empty bone lacunae were more likely to accumulate ox-LDL than LDL (Figure 2A). As shown in Figure 2B, the percentage of empty bone lacunae of ox-LDL-positive lacunae in the necrotic region was significantly greater than that of LDL-positive lacunae (*P* < 0.001). The percentage of empty lacunae was significantly correlated with the percentage of ox-LDL-positive lacunae (*P* < 0.01) and the IRS for ox-LDL (*P* < 0.005, Figure 2D), but not with LDL accumulation (*P* = 0.743 for LDL-positive lacunae and *P* = 0.622 for IRS, Figure 2C).

### Decreased blood supply in the necrotic region was correlated with LDL/ox-LDL accumulation.

**Fig. 3:**
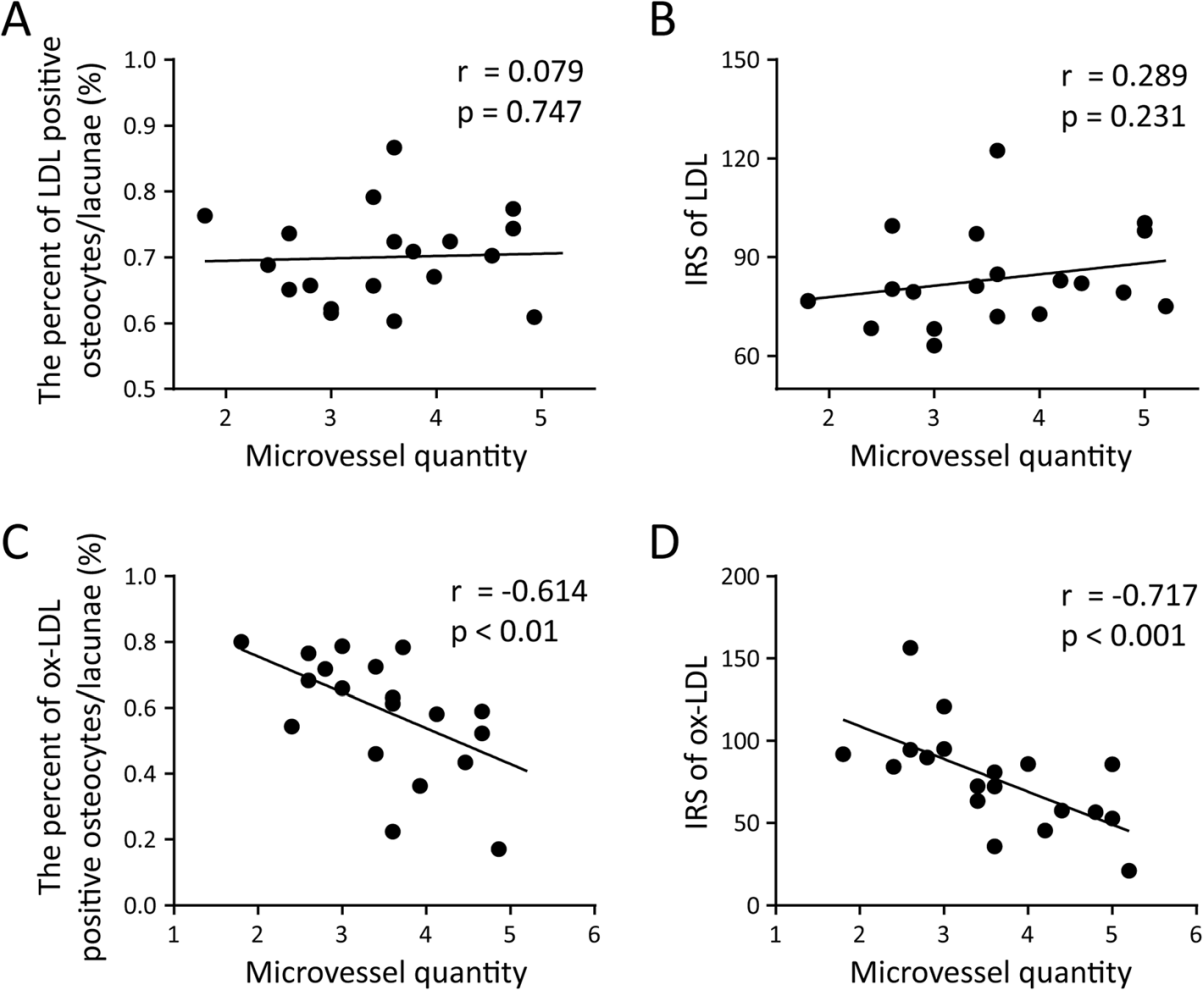
Correlation analysis between microvessel density and LDL/ox-LDL accumulation in the necrotic region. Correlation between the percentage of LDL-positive osteocytes/lacunae (**A**) and IRS for LDL (**B**) with the quantity of microvessels per field in the necrotic region. Correlation between the percentage of ox-LDL-positive osteocytes/lacunae (**C**) and IRS for ox-LDL (**D**) with the quantity of microvessels per field in the necrotic region. Ten random fields (400×) in each specimen were observed and calculated for analysis. Spearman’s correlation coefficients are used for correlation analysis

Correlation analysis was conducted to examine which factors were correlated with LDL/ox-LDL accumulation (Table 1). LDL and ox-LDL accumulation were not correlated with the patients’ age, body mass index, or risk factors. Furthermore, there was no correlation between LDL accumulation and ox-LDL accumulation. The Association Research Circulation Osseous (ARCO) stage of ONFH was positively correlated with the percentage of ox-LDL-positive osteocytes/lacunae (*P* < 0.05) but not the percentage of LDL-positive osteocytes/lacunae (*P* > 0.05). Although the density of medullary microvessels was not correlated with LDL accumulation (Figures 3A and B), the microvessel density was significantly and negatively correlated with the percentage of ox-LDL-positive osteocytes/lacunae (*P* < 0.01) and with the IRS for ox-LDL (*P* < 0.001, Figures 3C and D).

**Table 1:** Potential correlation factors associated with LDL/ox-LDL accumulation in the necrotic region

Correlation factors		Age	BMI^***a***^ (kg/m^**2**^)	Risk factors			ARCO stage (2019)				MVD^***c***^	MVMVD^***d***^	Percentage of LDL-positive lacunae	IRS^**e**^ for LDL
				Trauma	GC^*b*^	Alcohol	II	IIIa	IIIb	IV				
**LDL**	Percentage of positive lacunae (%)	\	\	67±2	71±3	70±2	64±3	71±4	67±5	71±2	\	\	\	\
	*P*-value	>0.05	>0.05	>0.05			>0.05				>0.05	>0.05	\	\
	IRS	\	\	72±3	88±6	83±3	84±1	89±7	70±2	82±5	\	\	\	\
	*P*-value	>0.05	>0.05	>0.05			>0.05				>0.05	>0.05	\	\
**ox-LDL**	Percentage of positive lacunae (%)	\	\	64±4	50±8	63±5	49±13	46±9	65±1	67±4	\	\	\	\
	*P*-value	>0.05	>0.05	>0.05			**<0.05**				**<0.01**	>0.05	>0.05	>0.05
	IRS	\	\	92±10	71±15	75±7	63±18	70±20	97±24	80±5	\	\	\	\
	*P*-value	>0.05	>0.05	>0.05			>0.05				**<0.001**	>0.05	>0.05	>0.05

^a^*BMI* body mass index, ^b^ *GC* glucocorticoid, ^c^ *MVD* microvessel density, ^d^ *MVMVD* mean value of microvessel diameter, ^e^ *IRS* immunoreactive score.

Data are represented as the mean ± SD

### Ox-LDL had greater effects on decreasing cell viability and enhancing apoptosis than LDL in MLO-Y4s.

**Fig. 4:**
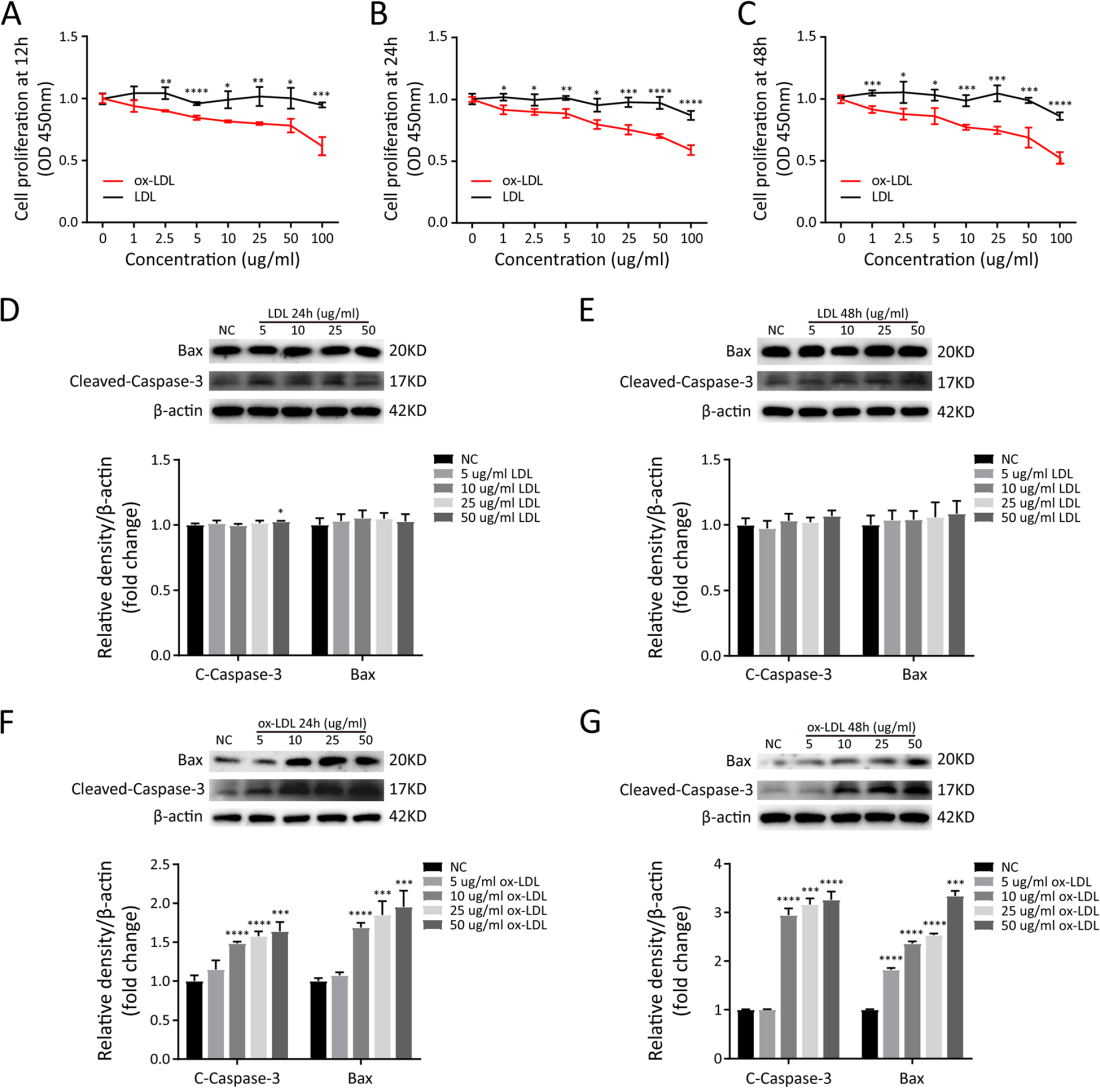
Effects of LDL and ox-LDL on cell viability and apoptosis in MLO-Y4 cells. **A**, **B** and **C** Effects on cell viability in MLO-Y4 cells after incubation with LDL or ox-LDL for 12, 24, or 48h. **D** and **E** Western blotting and quantitative analysis of cell apoptosis in MLO-Y4 cells incubated with LDL for 24 or 48 h. **F** and **G** Western blotting and quantitative analysis of cell apoptosis in MLO-Y4 cells incubated with ox-LDL for 24 or 48 h. Data are represented as the mean ± SD of three independent experiments. Comparisons are made using Student’s *t* test. *P <0.05; **P <0.01; ***P <0.005; ****P <0.001

Figures 4A–C show that ox-LDL significantly decreased the viability of MLO-Y4s in dose- and time-dependent manners. By contrast, LDL hardly affected MLO-Y4 viability, with a small decrease in cell viability at the highest concentrations tested (50 and 100μg/mL). At concentrations below 50 μg/mL, LDL hardly induced apoptosis of MLO-Y4 (Figures 4D and E). Incubation with ox-LDL for 24 and 48h significantly up-regulated the levels of cleaved-caspase3 and Bax in MLO-Y4s in dose-dependent manners (Figures 4F and G). These results indicate that after incubation for 24 or 48h, ox-LDL tended to induce more severe apoptosis of MLO-Y4 cells than LDL.

### MLO-Y4 cells could internalise LDL and ox-LDL.

**Fig. 5:**
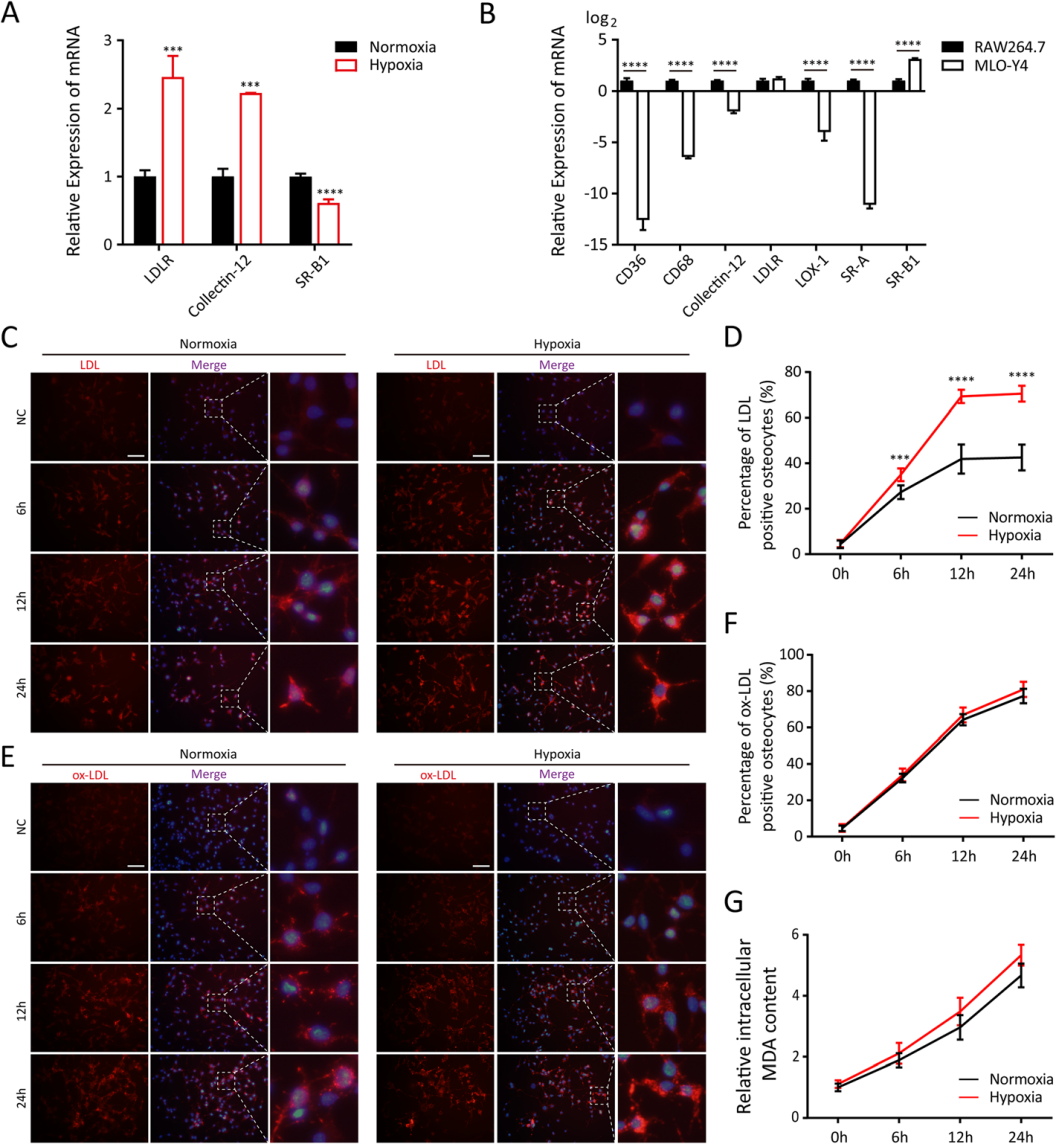
The cellular uptake of LDL and ox-LDL in MLO-Y4 cells in normoxic and hypoxic environments. **A** The relative expression of LDLR, collectin-12 and SR-B1 in MLO-Y4 cells in normoxic and hypoxic environments. **B** The relative expression of LDLR and main SRs in MLO-Y4 and RAW264.7 cells. **C** and **D** Immunofluorescence and quantitative statistics of LDL internalised by MLO-Y4 cells in normoxic and hypoxic environments. **E** and **F** Immunofluorescence and quantitative statistics of ox-LDL internalised by MLO-Y4 cells in normoxic and hypoxic environments. **G** The relative MDA content in MLO-Y4 cells after incubation with ox-LDL in normoxic and hypoxic environments. Data are represented as the mean ± SD of three independent experiments unless particularly stated, and the percentage of LDL/ox-LDL-positive cells were calculated from five random fields (200×). Comparisons are made using Student’s *t* test. ***P <0.005; ****P <0.001; scale bar = 100μm

qRT-PCR revealed that MLO-Y4 cells expressed the LDL receptor (LDLR) and scavenger receptors (SRs), including collectin-12 and SR-B1, which are responsible for binding to and internalising ox-LDL (Figures 5A and B). RAW 264.7 cells, used as a positive control, also expressed other SRs including LOX-1, SR-A, CD36, and CD68, which participate in the cellular uptake of ox-LDL, but these receptors were not expressed or were expressed at low levels in MLO-Y4s (Figure 5B). Immunofluorescence and quantitative analyses indicated that MLO-Y4 cells internalised LDL and ox-LDL after incubation for 6–24h (Figures 5C–F). The fluorescence intensity of LDL taken up by MLO-Y4 cells gradually increased up to 12h, but remained almost constant from 12 to 24h. By comparison, the amount of ox-LDL taken up by MLO-Y4 cells, as well as the intracellular MDA content after incubation with ox-LDL (Figure 5G), continued to increase for 24h.

### Hypoxia enhanced LDL uptake but did not significantly affect ox-LDL uptake by MLO-Y4 cells.

The immunofluorescence studies (Figures 5C and D) revealed that exposure to hypoxia significantly enhanced the cellular uptake of LDL in MLO-Y4 cells relative to the normoxic environment. However, hypoxia did not significantly affect ox-LDL uptake (Figures 5E and F). In the hypoxic environment, the amount of LDL internalised by MLO-Y4s peaked at 12h and remained constant, whereas the intracellular amount of ox-LDL continued to increase for 24h, similar to the results from MLO-Y4s cultured in the normoxic environment. The oxidative status of MLO-Y4s incubated with LDL or ox-LDL was evaluated by measuring the MDA content. Although the total intracellular MDA content continued to increase at 24h, hypoxia did not significantly affect intracellular MDA accumulation compared with normoxia (Figure 5G). Consistent with the immunofluorescence and MDA studies, qRT-PCR revealed that the expression of LDLR and collectin-12 were up-regulated, but the expression of SR-B1 was down-regulated by hypoxia (Figure 5A). Thus, hypoxia significantly enhanced the cellular uptake of LDL, but did not significantly affect ox-LDL internalisation.

### MLO-Y4 cells could oxidize LDL particles in a process that was enhanced by hypoxia.

**Fig. 6:**
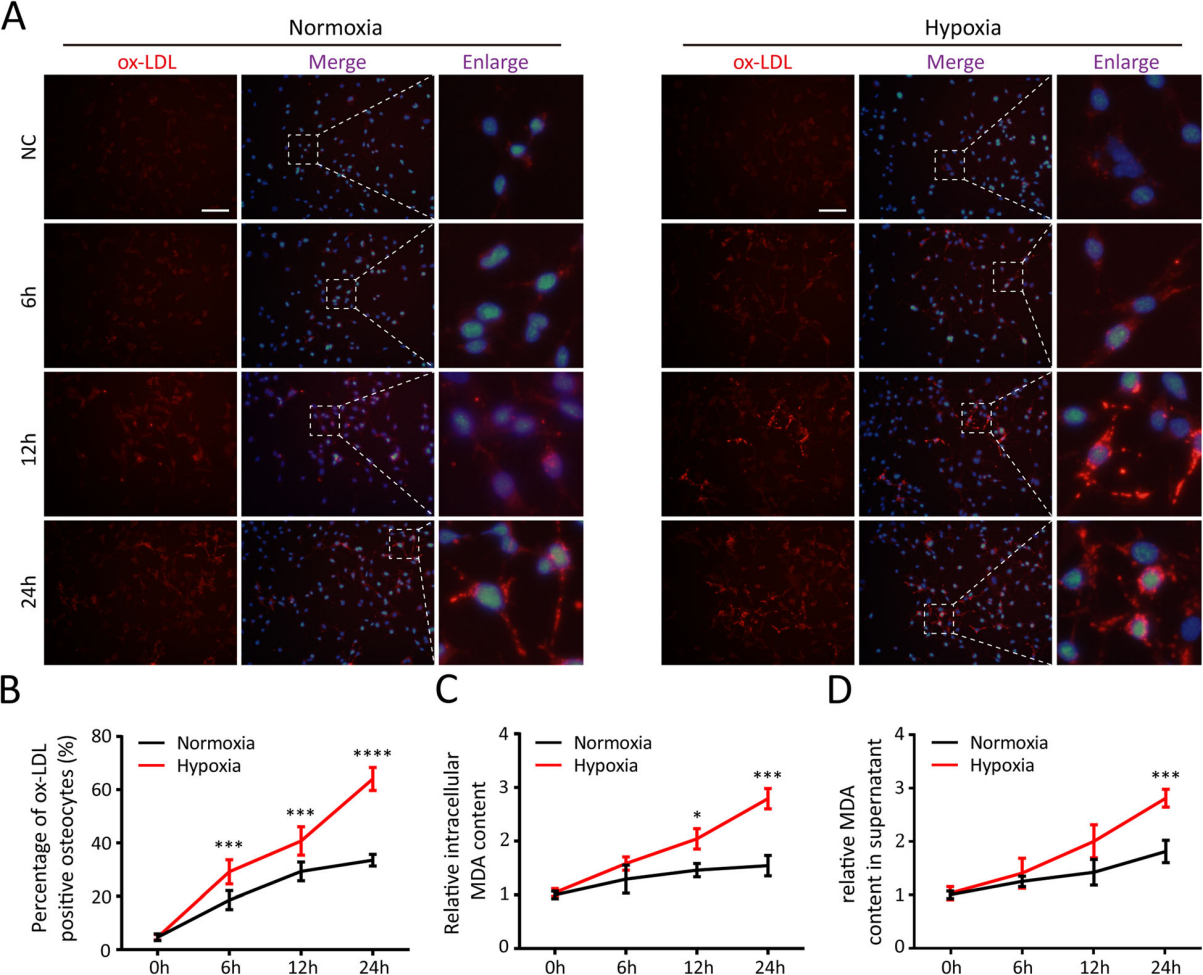
The LDL oxidative modification capability of MLO-Y4 was enhanced in hypoxic environment. **A** and **B** Immunofluorescence and quantitative statistics of ox-LDL oxidized from LDL by MLO-Y4 cells in normoxic and hypoxic environments. **C** The relative MDA content in MLO-Y4 cells after incubation with LDL in normoxic and hypoxic environments. **D** The relative MDA content in culture supernatant of MLO-Y4 cells after incubation with LDL in normoxic and hypoxic environments. Data are represented as the mean ± SD of three independent experiments unless particularly stated, and the percentage of ox-LDL-positive cells were calculated from five random fields (200×). Comparisons are made using Student’s *t* test. *P <0.05; ***P <0.005; ****P <0.001; scale bar = 100μm

Figures 6A and B show that the accumulation of ox-LDL in MLO-Y4 cells incubated with LDL increased in a time-dependent manner. Additionally, the MDA levels in MLO-Y4s and culture supernatant increased following incubation with LDL (Figures 6C and D). Thus, MLO-Y4 cells could oxidize LDL particles to ox-LDL. The increase of MDA and ox-LDL was slower after incubation with LDL for more than 12h.

Regarding the effects of hypoxia on oxidation of LDL, the percentage of ox-LDL-positive cells in the hypoxic environment increased significantly after incubation with LDL for 6, 12, or 24h (Figures 6A and B). After incubation with LDL for 24 h, the MDA levels in MLO-Y4s and culture supernatant were significantly greater in the hypoxic environment than in the normoxic environment (Figures 6C and D). Furthermore, unlike in the normoxic environment, the accumulation of ox-LDL and MDA increased rapidly in MLO-Y4 cells incubated with LDL for 12 to 24h.

## Discussion

Disordered lipid metabolism, including LDL metabolism, is a common pathological process in ONFH. However, there is limited knowledge about the local pathophysiological metabolism of LDL in the necrotic femoral head. To our knowledge, this is the first study to report the accumulation of LDL/ox-LDL in the necrotic femoral head.

The present study revealed that LDL and ox-LDL accumulated significantly in the necrotic region. Although cell death and lysis emptied the bone lacunae in dead trabeculae, the IHC studies indicated that most of the empty bone lacunae contained LDL and ox-LDL. However, the percentage of empty lacunae among ox-LDL-positive lacunae was significantly higher than that among LDL-positive lacunae. This suggests that the death of osteocytes is more likely to be associated with ox-LDL than with native LDL. Subsequent *in vitro* experiments revealed that ox-LDL decreased osteocyte viability and enhanced apoptosis in dose- and time-dependent manners, whereas LDL hardly affected the osteocytes. Because ox-LDL contains various toxic oxidized lipids, numerous studies have demonstrated that it triggers cellular oxidative stress [12], endoplasmic reticulum stress [13], biofilm dysfunction [14], lysosomal membrane damage [15, 16], mitochondrial dysfunction and apoptosis [17]. Recent studies have also suggested that ox-LDL may be a determinant of osteoporosis because it inhibits the differentiation of BMSCs to osteoblasts [18] and the osteoblast mineralization [19]. By comparison, ox-LDL promotes the formation of osteoclasts and enhances bone resorption [20], and disturbs the crosstalk between osteoblasts and osteoclast [21]. However, few studies have examined the pathophysiological effects of ox-LDL in osteocytes.

Although osteocyte apoptosis is a fundamental pathological mechanism of ONFH, the accumulation of ox-LDL may promote other mechanisms, including bone metabolism and microvessels disorders. In addition, ox-LDL may induce osteoblast apoptosis directly [15, 16]. Regarding microvessel disorders, there have been numerous studies showing that ox-LDL promotes inflammation, oxidative damage, and apoptosis in vascular endothelial cells [22–24]. Moreover, ox-LDL may impair the proliferation and network formation of endothelial progenitor cells, and induce their cell senescence and apoptosis [25, 26]. Ox-LDL also suppresses endothelial differentiation and induces apoptosis of bone marrow multipotent progenitor cells [27].

The internalisation of native LDL is mediated by LDLR, but this mainly involves several SRs, including LOX-1, SR-A, CD36, SR-BI, CD68, and collectin-12, after oxidation. Unlike LDL, the uptake of ox-LDL was not negatively regulated by intracellular cholesterol content, which readily causes intracellular ox-LDL accumulation [28]. Here, it was found that, after incubating osteocytes with LDL or ox-LDL at the same concentration, the intracellular content of LDL remained stable after 12h, but the accumulation of ox-LDL continued to increase for 24h. Because previous studies examining ox-LDL metabolism mainly used macrophages or vascular endothelial cells, it was assumed that LOX-1, SR-A, CD36, and SR-BI were the main SRs responsible for internalisation of ox-LDL [29, 30]. However, the present study revealed that collectin-12 and SR-B1 might be the primary SRs involved in ox-LDL uptake in osteocytes. It was also demonstrated that the osteocyte expressed high levels of LDLR and could internalise and oxidize native LDL. Similarly, it was reported that the LDLR expression in osteoblasts was high [31] and that they can also oxidize LDL [15]. Although osteoblasts may exacerbate the accumulation of ox-LDL, osteocytes are more likely to play a major role because they account for over 90% of bone cells. Although there have been several studies examining SR expression in osteoblasts and osteocytes, the results were controversial. For example, consistent with the present results, it was shown that SR-B1 was prominently expressed in MLO-Y4 cells and osteoblasts, whereas CD36 expression was low or undetectable [32]. By contrast, Brodeur et al. reported that SR-B1 and CD36 were expressed in osteoblast-like cell lines [33]. Similar to the present results, Kalajzic et al. detected low SR-A expression in osteoblasts, but a higher level of CD68, which increased during osteogenic differentiation [34]. The present study suggests that collectin-12, a newly-identified SR, may play a key role in ox-LDL metabolism in osteocytes.

Considering that the accumulation of LDL/ox-LDL did not differ significantly among traumatic, GC-associated, and alcohol-associated ONFH, this may be a common pathological process in ONFH. Thus, the negative correlation between ox-LDL accumulation and medullary blood supply in the necrotic region seems reasonable. Impaired blood supply creates a hypoxic environment for osteocytes in the necrotic region, a fundamental pathological change in ONFH. The present study revealed that hypoxia significantly enhanced the internalisation and oxidation of LDL, but did not significantly affect ox-LDL uptake. Hypoxia can induce the accumulation of lipids in various cells; in particular, hypoxia enhances LDL internalisation in smooth muscle and cancer cells [35, 36]. Furthermore, macrophages oxidize native LDL to a greater extent in hypoxic environments than in normoxic environments [37]. In osteoblasts and osteocytes, hypoxia enhances reactive oxygen specie generation and oxidative stress [38–40]. Imamura et al. reported that hypoxia could enhance lipid uptake and foam cell formation in macrophages treated with native LDL [41], which is similar to the results in osteocytes in the present study. Considering the large difference between the circulating concentrations of LDL and ox-LDL [42, 43], it is reasonable to speculate that the accumulation of ox-LDL in osteocytes is mainly due to increased internalisation and oxidation of native LDL, rather than direct uptake of ox-LDL.

### Comparisons with other studies and what does the current work add to the existing knowledge

Over the last decade, evidence been accumulating that ox-LDL is important in the pathogenesis of osteoarthritis, another common joint disease that often requires surgical treatment [44]. It was reported that ox-LDL accumulated in chondrocytes in the pathological process of osteoarthritis and that LOX-1-deficient mice were resistant to osteoarthritis induced by instability or age [45, 46]. Whereas, few researches have studied the pathophysiological role of ox-LDL in ONFH. This study showed that greater accumulation of LDL/ox-LDL was found in the necrotic regions compared with healthy regions. *In vitro* studies showed that ox-LDL, but not native LDL, might be responsible for aggravating osteocyte viability and enhancing apoptosis. Because the accumulation of ox-LDL was associated with a reduced blood supply, the present study also investigated the impact of hypoxia on LDL/ox-LDL metabolism *in vitro*. The enhanced internalisation and oxidation of LDL by osteocytes in the hypoxic environment could at least partially explain the increased accumulation of LDL/ox-LDL in the necrotic region.

Because it may be difficult to improve oxygen and blood supply without surgery, treatments that reduce the concentration and cellular uptake of LDL in the femoral head, as well as antioxidant therapies [19, 21], may be promising conservative methods to prevent or treat early stage ONFH. Lipoprotein(a), a variant LDL particle, plays key roles in atherosclerosis, cardiovascular events, and lipid disorders [47, 48]. It was reported that high blood lipoprotein(a) levels and low-molecular-weight lipoprotein(a) isoform were associated with increased risk of ONFH [49, 50]. Thus, more attention should be paid to lipoprotein(a) in lipid accumulation and ONFH, beyond LDL/ox-LDL. Intriguingly, lipoprotein(a) is also internalised via LDLR, and therapies aimed at reducing the content of LDLR in osteocytes or LDLR antibodies may also be beneficial by regulating lipoprotein(a) metabolism.

### Study strengths and limitations

To our knowledge, it is the first study to show that LDL and ox-LDL accumulated in the necrotic region and may participate in ONFH. Pathological and *in vitro* studies showed that ox-LDL could induce osteocyte apoptosis, and thus contributed to ONFH. The findings may shed new light on the prevention and treatment of ONFH. The main limitation is that the present study did not examine the relationship between the circulating LDL level and its level in the femoral head; this relationship is worth investigating in future studies. Because it is virtually impossible to obtain femoral head specimens in the initial stages of ONFH, the present study was unable to demonstrate whether the accumulation of LDL/ox-LDL plays a crucial role in the onset of ONFH or just involved in the pathological process after onset. Animal experiments are needed to answer this question in the future. Further *in vitro* studies are also needed to investigate the oxidation of LDL in osteocytes in detail. Another limitation is that the sample size might not be enough to compare the LDL/ox-LDL accumulation among different types of ONFH. Thus, there may not be sufficient power to reach a conclusion that the accumulation of LDL/ox-LDL is unrelated to the cause of ONFH. However, it can be concluded that the accumulation of LDL/ox-LDL is a common pathological process in ONFH, at least for ONFH associated with trauma, GC, and alcohol.

## Conclusions

This pilot study shows that the accumulation of LDL/ox-LDL in the femoral head participates in the pathological process of ONFH. Disordered LDL/ox-LDL metabolism in osteocytes induced by hypoxia could be a potential pathologic mechanism. With a lack of conservation therapies to slow down the pathological process of ONFH, the present study has identified a promising therapeutic target. Strategies that reduce the cellular uptake of LDL in the femoral head, perhaps by regulating LDLR levels or using LDLR antibodies, combined with antioxidants, merit further *in vivo* and clinical studies.

## Supplementary Information

**Additional file 1.**

## Data Availability

All data for this study are contained within this article and in the supplemental data.

## Abbreviations

ARCO: Association Research Circulation Osseous
BMSC: Bone marrow stromal cell
BSA: Bovine serum albumin
CCK-8: Cell Counting Kit-8
CS: Calf serum
FBS: Fetal bovine serum
GC: Glucocorticoid
IHC: Immunohistochemistry
IRS: Immunoreactive score
LDL: Low-density lipoprotein
LDLR: Low-density lipoprotein receptor
MDA: Malondialdehyde
MLO-Y4: Murine long-bone osteocyte Y4
ONFH: Osteonecrosis of the femoral head
ox-LDL: Oxidized low-density lipoprotein
qRT-PCR: Quantitative real-time polymerase chain reaction
SD: Standard deviation
THA: Total hip arthroplasty
