# Supplementary material for: Accumulation of LDL/ox-LDL in the necrotic region participates in osteonecrosis of the femoral head: a pathological and in vitro study
Source: Lipids Health Dis. 2021 Nov 25;20:167. doi: 10.1186/s12944-021-01601-x (PMC8620162; doi:10.1186/s12944-021-01601-x)
Supplement: Supplementary file 1 — Additional file 1. [file 12944_2021_1601_MOESM1_ESM.docx]

**Supplemental material**

**The inclusion and exclusion criteria of patients in the present study**

**Inclusion criteria:**

1. Age above 18 and no limitation of gender;

2. Patients who have underwent total hip arthroplasty or core decompression with bone grafting;

3. Osteonecrosis of the femoral head confirmed by pathological diagnosis;

**Exclusion criteria:**

1. Accompanied with severe hip malformation, developmental dysplasia of the hip, dislocation or subluxation of the hip;

2. Accompanied with hip tumor, hip infection, or hematological diseases;

3. Patients who had underwent total hip arthroplasty, hemiarthroplasty, other hip-preserving surgeries, bone biopsy, or extracorporeal shock wave therapy on the same side before this operation;

4. Patients who were taking lipid regulators.

**Supplemental Table 1.** **Clinical characteristics of the patients with ONFH**

| Patient | Age | Gender | Operation side | BMI^a^ (kg/m^2^) | Risk factors | ARCO stage (2019) | Chronic diseases | Long-term medication |
| --- | --- | --- | --- | --- | --- | --- | --- | --- |
| 1 | 63 | Male | Left | 24 | Trauma | IV | \ | \ |
| 2 | 47 | Male | Right | 26 | Trauma | III b | \ | \ |
| 3 | 73 | Male | Right | 28 | Trauma | III b | Hypertension, Parkinson | Valsartan, Spirolactone, Madopar, Pramipexole |
| 4 | 61 | Female | Left | 24 | Trauma | III a | Osteoporosis, Supraventricular tachycardia | Calcitriol, Zoledronic acid |
| 5 | 49 | Female | Right | 24 | GC^b^ | III a | Asthma | Budesonide, Montelukast |
| 6 | 41 | Female | Right | 19 | GC | IV | SLE^c^, Hypertension | Medrol, Hydroxychloroquine, Mycophenolate mofetil, Candesartan |
| 7 | 67 | Female | Left | 21 | GC | IV | ANCA^d^-mediated glomerulonephritis, ANCA-mediated vasculitis, Hypertension | Medrol |
| 8 | 21 | Male | Right | 19 | GC | III a | Nephrotic syndrome | Prednisone acetate |
| 9 | 22 | Male | Right | 25 | GC | III a | \ | \ |
| 10 | 33 | Female | Left | 30 | GC | II | \ | \ |
| 11 | 35 | Male | Right | 19 | GC | III a | Kidney transplant, Hypertension | Medrol, Ciclosporin, Mycophenolate mofetil, Nifedipine, Losartan, Metoprolol, Diltiazem |
| 12 | 29 | Female | Right | 28 | GC | II | Sicca syndrome | Prednisone, Ciclosporin, Hydroxychloroquine, Calcitriol |
| 13 | 63 | Male | Left | 27 | Alcohol | IV | Hypertension | Amlodipine besylate tablet |
| 14 | 58 | Male | Left | 22 | Alcohol | III a | \ | \ |
| 15 | 70 | Male | Left | 21 | Alcohol | IV | Epilepsy | Carbamazepine, Clonazepam |
| 16 | 46 | Male | Left | 19 | Alcohol | IV | \ | \ |
| 17 | 72 | Female | Left | 22 | Alcohol | IV | \ | \ |
| 18 | 47 | Male | Left | 26 | Alcohol | IV | \ | \ |
| 19 | 47 | Male | Right | 31 | Alcohol | IV | \ | \ |

1. BMI: body mass index, b. GC: glucocorticoid, c. SLE: systemic lupus erythematosus. d: ANCA: antineutrophil cytoplasmic autoantibody.

**Supplemental Table 2 Primer Sequences for qRT-PCR**

| **Primer** |  | **Nucleotide sequence** |
| --- | --- | --- |
| **CD36** | Forward | 5'-CTTTGAAAGAACTCTTGTGGGG-3' |
|  | Reverse | 5'-GTCTGTGCCATTAATCATGTCG-3' |
| **CD68** | Forward | 5'-GAAATGTCACAGTTCACACCAG-3' |
|  | Reverse | 5'-GGATCTTGGACTAGTAGCAGTG-3' |
| **collectin-12** | Forward | 5'-CACGGCAAATTCAACAGCACAGTC-3' |
|  | Reverse | 5'-CACAACATATTCAGCACCAGCATCAC-3' |
| **LDLR** | Forward | 5'-CAGAAGTCGACACTGTACTGAC-3' |
|  | Reverse | 5'-AAGATGGACAGGAACCTCATAC-3' |
| **LOX-1** | Forward | 5'-TGAAGCCTGCGAATGACGAG-3' |
|  | Reverse | 5'-GTCACTGACAACACCCAGGCAGAG-3' |
| **SR-A** | Forward | 5'-GAATTCACTGGATGCAATCTCC-3' |
|  | Reverse | 5'-TGTTGCTTTGCTGTAGATTCAC-3' |
| **SR-B1** | Forward | 5'-AACATCACCTTCAATGACAACG-3' |
|  | Reverse | 5'-ACCAAGATGTTAGGCAGTACAA-3' |
